# Eco-friendly spectrophotometric approach for the determination of anti-diabetic drugs in fixed-dose formulation together with metformin’s toxic impurity: comprehensive method assessment

**DOI:** 10.1038/s41598-026-38952-3

**Published:** 2026-03-21

**Authors:** Doaa G. Mohamed, Maha M. Abdelrahman, Amal B. Ahmed, Maimana A. Magdy

**Affiliations:** 1 Bachelor Of Pharmacy, Postgraduate study at Pharmaceutical Analytical Chemistry Department, Faculty of Pharmacy, Beni-Suef University, Alshaheed Shehata Ahmad Hegazy St, Beni-Suef, 62514 Egypt; 2 Pharmaceutical Analytical Chemistry Department, Faculty of Pharmacy, Beni-Suef University, Alshaheed Shehata Ahmad Hegazy St, Beni-Suef, 62514 Egypt; 3 Pharmaceutical Chemistry Department, Faculty of Pharmacy, Nahda University, Sharq El-Nile, Beni-Suef, 62511 Egypt

**Keywords:** Saxagliptin, Metformin, Melamine, Spectrophotometric method, Ratio difference, First derivative of ratio spectra, Chemistry, Environmental sciences

## Abstract

Environmentally benign and straightforward spectrophotometric methods were developed and validated for the simultaneous determination of saxagliptin (SAX), metformin (MET), and the pharmacopeial impurity of MET, melamine (MEL), in bulk powder and pharmaceutical formulations. These proposed approaches provide reliable, low-cost, and accessible alternatives to conventional chromatographic techniques, which often require complex instrumentation, extended analysis times, and significant solvent consumption. Two complementary spectrophotometric methods were established. Method A, the ratio-difference approach, utilized MEL as a divisor for the quantification of SAX, while SAX served as a divisor for determining MET and MEL. Method B, based on the first derivative of ratio spectra, applied MEL as a divisor under optimized conditions (scaling factor = 10, Δλ = 4 nm). However, MEL could not be quantified by Method B because its derivative ratio signals in the selected spectral region were too weak and overlapped extensively with those of SAX and MET, preventing the establishment of a reliable linear calibration curve for this impurity. The methods exhibited linearity over the ranges of 5–90 µg/mL for SAX, 1–40 µg/mL for MET, and 0.5–10 µg/mL for MEL. Validation in accordance with ICH Q2(R1) guidelines confirmed their accuracy, precision, selectivity, and specificity. The results obtained by the proposed methods were statistically compared with those of a reported RP‑HPLC method, confirming the reliability of the proposed procedures. The environmental impact was assessed using the principles of green analytical chemistry (GAC), which incorporates sustainability by promoting methods that limit hazardous reagents, reduce energy consumption, and minimize waste while maintaining suitable analytical performance. Holistic evaluation tools such as the analytical greenness metric (AGREE), the green analytical procedure index (GAPI), the national environmental methods index (NEMI), the blue applicability grade index (BAGI), and the RGB 12 model enable simultaneous assessment of analytical quality, environmental impact, and practical applicability. These tools foster the development of “white” analytical chemistry and complement emerging indices like the environmental and practical performance index (EPPI).Overall, these spectrophotometric approaches represent sustainable, efficient, and accessible analytical alternatives for the routine quality control of Saxagliptin and Metformin in combined pharmaceutical formulations.

**Supplementary Information:**

The online version contains supplementary material available at 10.1038/s41598-026-38952-3.

## Introduction

**Fig. 1:**
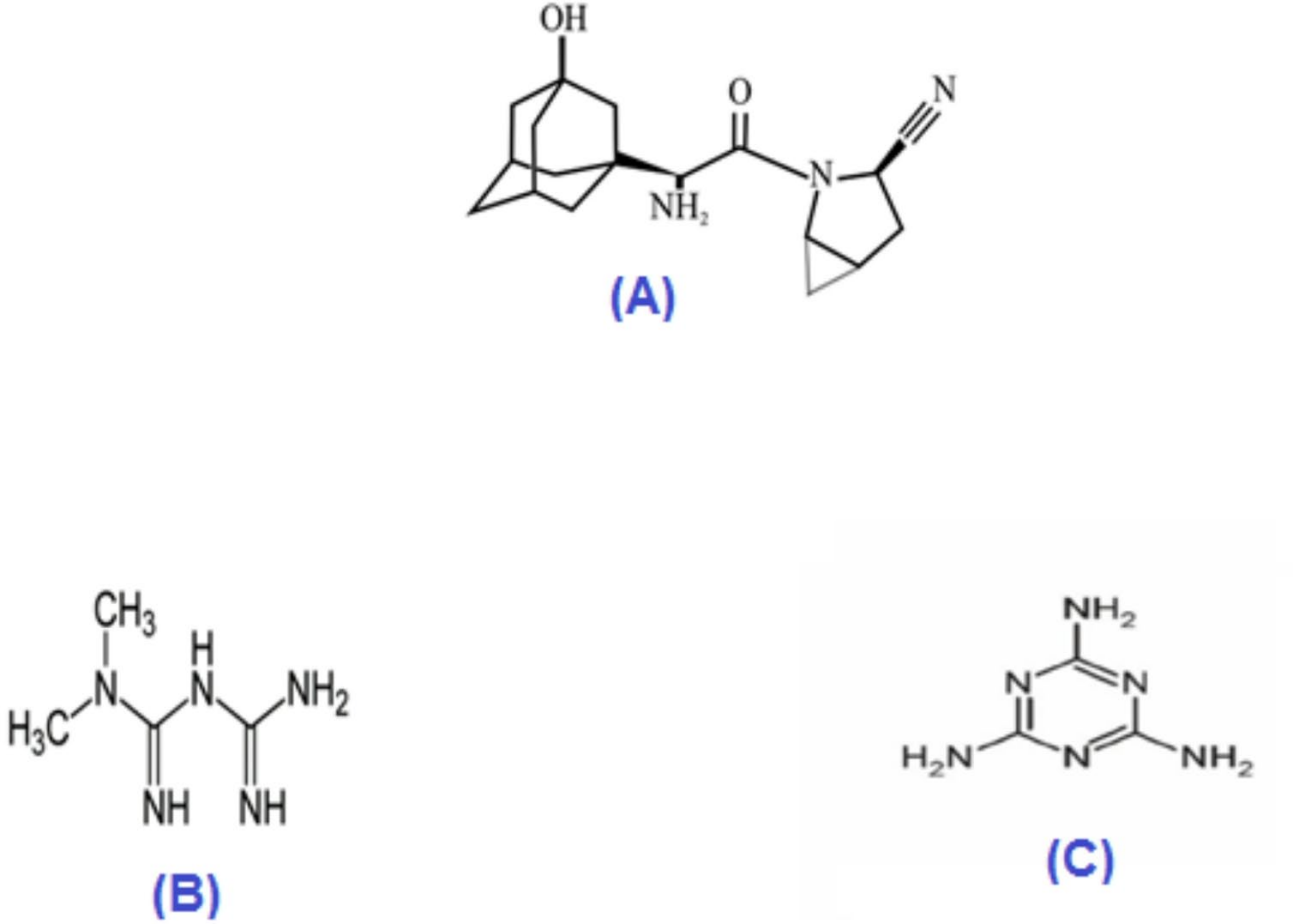
The chemical structure of (**a**) saxagliptin, (**b**) metformin, and (**c**) melamine.

The chemical name for the unofficial drug saxagliptin (SAX) is (1S,3S,5S)-2-[(2S)-2-amino-2-(3-hydroxy-1-adamantyl)acetyl]-2-azabicyclohexane-3-carbonitrile^1^, Fig. 1a. Saxagliptin HCl is an oral hypoglycemic agent that belongs to the dipeptidyl peptidase-4 (DPP-4) inhibitor class of drugs, is applied to the management of type II diabetes either alone or in combination with other medications^2,3^.

1,1-Dimethylbiguanide hydrochloride is the chemical name for metformin (MET)^1^, Fig. 1b. It is an oral anti-diabetic medication that increases the susceptibility of peripheral tissues and the liver to insulin without seriously impairing lactic acidosis. It is the first-line medication for type II diabetes mellitus and is official in The European Pharmacopoeia^4^, Indian Pharmacopoeia^5^, and United States Pharmacopoeia^6^.

The European Pharmacopoeia^4^ and the United States Pharmacopoeia^6^ list melamine (MEL), which is chemically known as 1,3,5-triazine-2,4,6-triamine^7^, Fig. 1c, as a related chemical and primary impurity of MET. Depending on the method of exposure, MEL toxicity may cause skin, eye, or lung irritation by inhalation or direct skin contact. Renal failure, kidney damage, and ultimately death are further consequences of prolonged exposure^8^. As a toxic process-related impurity, melamine is subject to strict controls in pharmaceuticals and food, with permissible levels generally in the low ppm range or below. For example, pharmacopeial standards for metformin restrict melamine to ≤ 0.01% (≈100 ppm), while food safety limits based on WHO guidance can be as low as 1–2.5 ppm in infant formula. These stringent regulations highlight the significant toxicological and regulatory risks of melamine contamination, necessitating highly sensitive analytical methods for reliable trace-level detection.

An extensive examination of previous academic literature has revealed that analysis of SAX and MET as a combination has been achieved by several analytical methods, such as spectrophotometry^9,10^, RP-HPLC^11–23^, and UPLC approaches^24^, either in pure form or in their pharmaceutical formulations. Different approaches were developed for the determination of MET in the presence of its impurities, including MEL as the primary impurity of MET. These approaches comprise a spectrophotometric method^25^ and chromatographic methods^26–30^. Only chromatographic HPLC and TLC methods have been reported for determining SAX and MET when MEL is present^31^.

Several analytical procedures have been reported for the determination of SAX and MET, including UV–VIS spectrophotometry, RP-HPLC, and UPLC. Conventional spectrophotometric methods for SAX/MET mixtures are generally simple and inexpensive but suffer from limited selectivity due to extensive spectral overlap and typically do not address the potential presence of the toxic impurity melamine (MEL). Chromatographic methods, on the other hand, provide high sensitivity and resolution; some recent HPLC procedures can quantify MET in the presence of MEL. However, they usually require relatively large volumes of organic mobile phases, longer analysis times, and higher operating costs and are seldom evaluated using modern green metrics. Moreover, most available methods focus either on the active ingredients or on MET–MEL alone, rather than enabling the simultaneous assay of SAX, MET, and MEL in a single run. Consequently, there is a need for a simple, low-solvent spectrophotometric strategy that is capable of selectively determining SAX and MET in their combined dosage form while also monitoring MEL as a hazardous impurity and being critically assessed in terms of greenness and practical applicability. This is the objective of the present work. The primary aim of this study is to develop and validate simple, economical, and environmentally sustainable spectrophotometric methods for the simultaneous determination of saxagliptin (SAX) and metformin (MET) in their combined dosage forms, raw materials, and in the presence of the pharmacopeial impurity of MET, melamine (MEL). The proposed ratio-difference and first-derivative spectrophotometric techniques offer distinct advantages over conventional chromatographic methods, including minimal solvent consumption, reduced waste generation, lower operating costs, and the elimination of the need for expensive or complex instrumentation. These approaches enable rapid analysis with high sensitivity and selectivity, making them particularly suited for laboratories with limited resources. The developed methods were validated in accordance with the International Council for Harmonization (ICH) guidelines^32^, confirming their accuracy, precision, and robustness. Additionally, greenness and blueness evaluations and the RGB 12 algorithm demonstrated that the proposed procedures have a low environmental footprint and strong compliance with sustainability and practicality criteria. Owing to their simplicity, affordability, and eco-friendliness, these spectrophotometric methods are highly suitable for routine quality-control applications in pharmaceutical analysis. Green analytical chemistry (GAC) extends the principles of green chemistry to analytical measurements by promoting methods that minimize the use of hazardous reagents, reduce energy consumption, and generate as little waste as possible while maintaining acceptable analytical performance. In this context, spectrophotometric and other low‑resource techniques are increasingly explored as sustainable alternatives to chromatographic procedures, which often require large volumes of organic solvents, long run times, and sophisticated instrumentation. Recent holistic metrics, such as the analytical greenness metric approach (AGREE), green analytical procedure index (GAPI), national environmental method index (NEMI), along with the blue applicability grade index (BAGI) and RGB 12 algorithms, evaluate analytical methods simultaneously in terms of analytical quality (red), environmental profile (green), and practical or economic aspects (blue), thereby supporting the development of so‑called “white” analytical chemistry^33–36^.

## Experimental

### Instrumental

A double-beam UV–visible spectrophotometer (SHIMADZU 1800 PC Series Spectrophotometer, Japan) with a 1.0 cm quartz cell and an attached IBM-compatible computer (Model UV-1601 PC) was used. All absorbance measurements and data analysis were performed using UV-PC Personal Spectroscopy software (version 3.7). Spectral measurements were recorded over the 200–400 nm range at a fast scan speed, with a spectral bandwidth of 1.0 nm and a data interval of 0.1 nm to optimize resolution and the signal-to-noise ratio. These settings ensured baseline smoothness (noise < 0.001 AU) and reproducible peak resolution.

### Materials

#### Pure standards

High-purity reference standards were sourced as follows: Metformin (MET, certified purity 99.88%) was kindly provided by Sigma Pharmaceuticals Industries (El Monofeya, Egypt). Saxagliptin (SAX, certified purity 99.97%) was supplied by Novartis Pharma (Cairo, Egypt). Melamine (MEL, certified purity 95.56%) was obtained from Sigma-Aldrich (Germany).

#### Pharmaceutical formulation

A commercial batch (No. 3932500) of Kombiglyze XR® tablets, manufactured by AstraZeneca, was obtained from a local pharmacy in Egypt. Each tablet is labeled to contain 1000 mg of metformin hydrochloride and 5 mg of saxagliptin hydrochloride.

#### Chemicals and reagents

Reagents and chemicals of analytical grade were used without further purification. Analytical-grade methanol (AnalaR, CDH, India) and deionized water (SEDICO Pharmaceuticals, Cairo, Egypt) were employed.

### Prepared solutions

Standard stock solutions 1000 µg/mL (1 mg/mL) were prepared by precisely weighing 100 mg of each pure standard into separate 100-mL volumetric flasks and then diluted to volume with methanol to each flask. These stock solutions were then further diluted with deionized water to obtain the required standard working solutions (100 µg/mL).

Laboratory-prepared mixtures containing different ratios of MET, SAX, and MEL were prepared using their standard working solutions (100 µg/mL), with water as the final diluent.

Stock standard solutions of SAX, MET, and MEL were stored in tightly closed amber volumetric flasks at 4 ± 2 °C and were found to be stable for at least two weeks, as no significant change in absorbance or assay result was observed (*p* > 0.05). Daily working solutions were prepared by appropriate dilution with water and used within 24 h at room temperature to avoid potential degradation or evaporation.

### Methods

#### Linearity and construction of the calibration curves

To achieve concentration ranges of 5–90 µg/mL for SAX, 1–40 µg/mL for MET, and 0.5–10 µg/mL for MEL, appropriate aliquots were taken from the corresponding working solutions (100 µg/mL) and transferred into separate 10-mL volumetric flasks. Each flask was then diluted to volume with deionized water. The absorption spectra of the resulting solutions were recorded using a spectrophotometer against a water blank.

##### Ratio difference spectrophotometric method

To select a suitable wavelength pair for the ratio-difference method, the spectra of the studied components were first assessed. For the determination of SAX, a standard spectrum of 8 µg/mL MEL was used as the divisor. The procedure involved calculating the difference in peak amplitudes of the ratio spectra of SAX between the two specific wavelengths of 243.2 nm and 253.8 nm. Conversely, a standard spectrum of 7 µg/mL SAX served as the divisor for determining MET and MEL. This involved measuring the difference in peak amplitudes at the wavelength pairs 220.7 and 234.6 nm for MET, and 206.9 and 220.0 nm for MEL, respectively. The regression equations were subsequently calculated by correlating these amplitude differences with the corresponding analyte concentrations^37^.

##### First derivative of the ratio spectra spectrophotometric method

A standard spectrum of 5 µg/mL MEL was used as a divisor to obtain the ratio spectra for SAX and MET. Next, the first derivative of these ratio spectra was generated using the instrumental parameters of a scaling factor of 10 and Δλ = 4 nm. The peak amplitudes of the resulting derivative spectra were then measured at 226.8 nm for SAX and at 248.2 nm for MET. Regression equations were constructed by plotting the measured peak amplitudes at each selected wavelength against the corresponding drug concentration.

#### Application to pharmaceutical formulation (Kombiglyze XR®)

Ten Kombiglyze XR® tablets were accurately weighed, ground into a fine powder, and transferred into a 100-mL volumetric flask. A portion of the powder was dissolved in methanol, and the mixture was sonicated for 20 min before being diluted to volume with methanol. After filtration through a 0.45-μm membrane filter, the resulting stock solution corresponded to a nominal concentration of 20 µg/mL for MET and 0.1 µg/mL for SAX, maintaining the formulation’s 200:1 (MET:SAX) ratio. Direct determination of SAX in this solution was not feasible, as its native concentration (~ 0.1 µg/mL) was below the method’s limit of quantification (LOQ) and linear range. Therefore, a standard addition approach was employed to accurately assess method performance in the pharmaceutical matrix. The prepared tablet solution was spiked with 5 µg/mL of pure SAX (from a 100 µg/mL stock), yielding a final SAX concentration of 5.1 µg/mL, which fell within the measurable range of the proposed spectrophotometric methods. Recovery studies confirmed negligible matrix interference. The mean recoveries (± RSD) for the ratio-difference method were 101.96 ± 0.86% for SAX and 101.72 ± 0.80% for MET. For the first-derivative ratio method, recoveries were 101.25 ± 1.25% for SAX and 98.82 ± 0.39% for MET. All results were within the ICH Q2(R1) acceptance limits of 98–102%, with %RSD values below 2%, confirming the method’s accuracy and precision in the presence of excipients. The practical, matrix-specific LOQ for SAX was determined to be 0.8 µg/mL, while the LOQ for MET remained 0.25 µg/mL, reflecting the method’s achievable sensitivity in the tablet formulation.

## Results and discussion

**Fig. 2:**
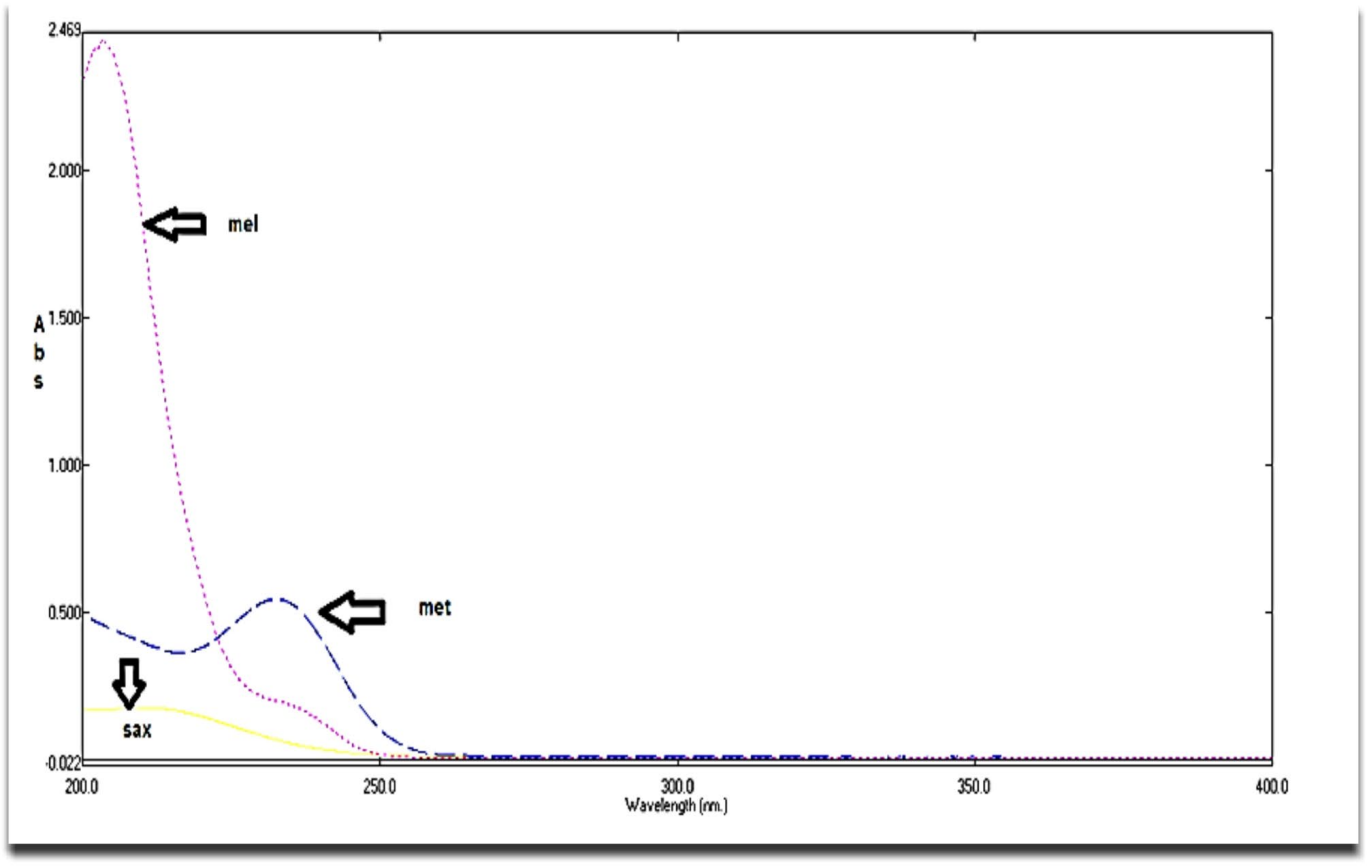
Zero‑order UV absorption spectra of saxagliptin (SAX, 7µg/mL), metformin (MET, 10 µg/mL), and melamine (MEL, 5 µg/mL) in the selected solvent system over 200–400 nm. The figure highlights the extensive spectral overlap among the three components.

Spectrophotometry is a cornerstone technique in pharmaceutical analysis due to its inherent efficient, economical, precise, and adaptability. These attributes make spectrophotometric methods reliable and practical for pharmaceutical applications, including stability-indicating assays in the presence of degradants or impurities^38,39^.As shown in Fig. 2, the UV absorption spectra of SAX, MET, and MEL exhibit significant spectral overlap, making direct determination of these compounds challenging. Consequently, from an analytical perspective, it was essential to develop a spectrophotometric approach capable of resolving such overlapping spectra without requiring prior separation steps.

### Method development and optimization

Several parameters can influence the effectiveness of the proposed techniques, including solvent selection, divisor concentration, and the chosen wavelength pairs. Therefore, a systematic optimization was conducted. First, various solvents including methanol, ethanol, water, 0.1 N HCl, and 0.1 N NaOH were evaluated. Water demonstrated superior performance, yielding steeper calibration slopes, lower LOD/LOQ values, and reduced blank noise compared to organic or acidic/alkaline media. Moreover, the recoveries and %RSD values obtained with water were superior or comparable to those in other solvents, while aligning with green chemistry principles by eliminating the need for bulk organic solvents. Second, divisor concentrations were optimized empirically by evaluating the resulting ratio spectra and calibration data. Excessively low concentrations increased noise, while high values compressed the signal and reduced sensitivity. The selected concentrations 8 µg/mL MEL as the divisor for SAX, and 7 µg/mL SAX as the divisor for MET and MEL produced the smoothest ratio spectra, highest correlation coefficients, and lowest %RSD, and were therefore deemed optimal. Finally, the wavelength pairs were carefully chosen based on spectral behavior and validation statistics. Regions were identified where the analyte exhibited a pronounced concentration-dependent amplitude change while the interfering components remained nearly constant, effectively canceling their contribution in the amplitude difference. Among the pairs evaluated, 243.2/253.8 nm for SAX, 220.7/234.6 nm for MET, and 206.9/220.0 nm for MEL provided the best combination of sensitivity, linearity, and minimal interference, and were thus selected for the final methods.

#### Ratio difference spectrophotometric method

**Fig. 3:**
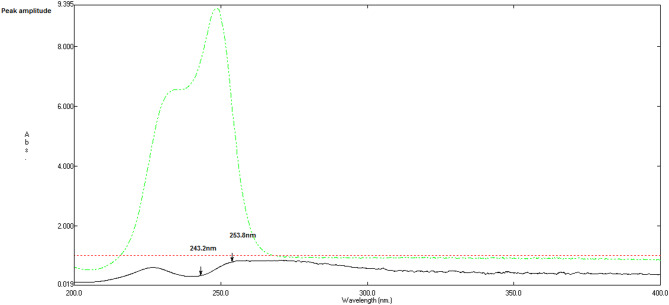
Ratio spectra of metformin HCl (MET, 20 µg/mL----), melamine (MEL, 6 µg/mL……), and saxagliptin (SAX, 15 µg/mL———) obtained by dividing each zero‑order spectrum by that of MEL (8 µg/mL) using water as solvent. The plot illustrates how use of MEL as a divisor generates well‑resolved ratio spectra in which SAX exhibits a characteristic profile, enabling its selective quantification by the ratio‑difference method in the presence of MET and MEL.

**Fig. 4:**
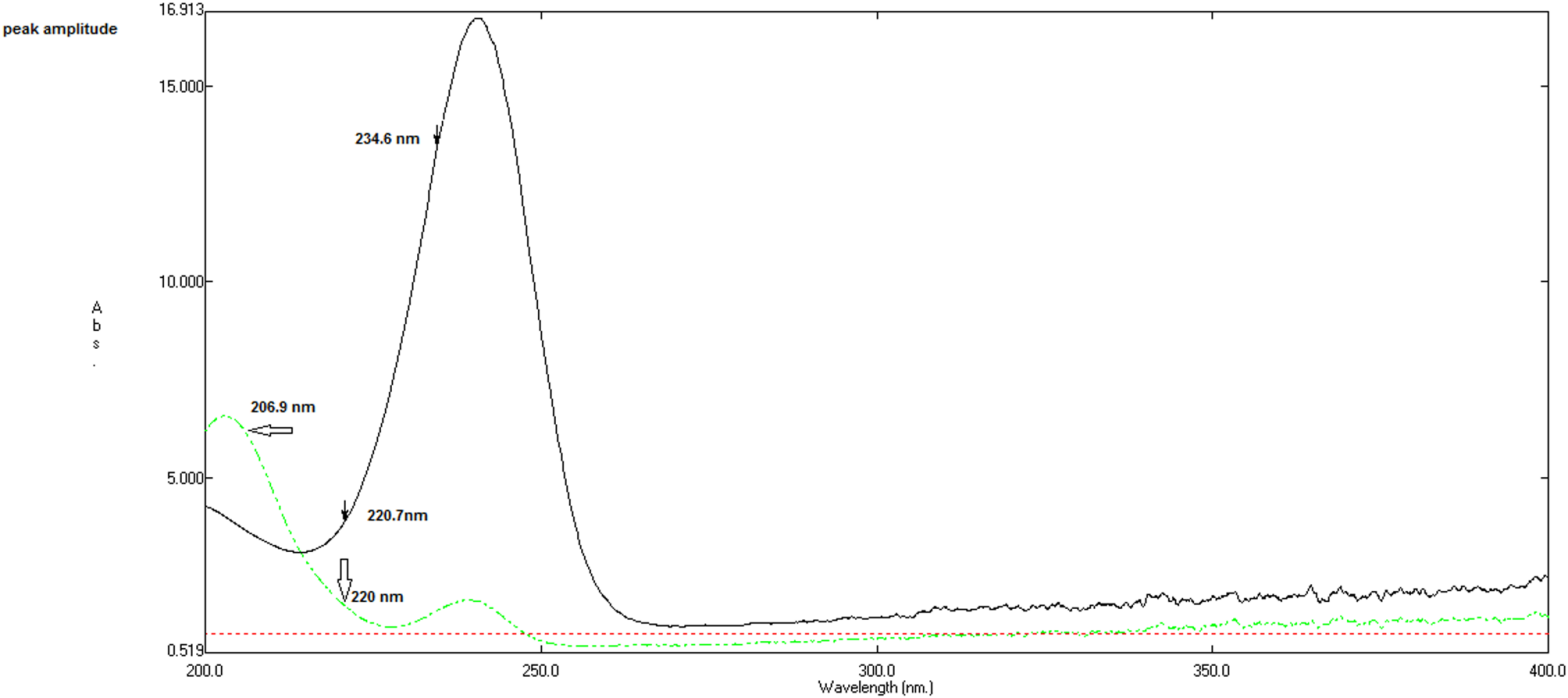
Ratio spectra of metformin HCl (MET, 10 µg/mL, ———), melamine (MEL, 5 µg/mL, ----), and saxagliptin (SAX, 20 µg/mL, ……) obtained by dividing each zero‑order spectrum by that of SAX (7 µg/mL) using water as the solvent. this figure demonstrates how using SAX as a divisor generates distinct ratio profiles for MET and MEL.

The development of the ratio-difference method focused on optimizing conditions to maximize spectral contrast among overlapping components while minimizing interference. The selection of the divisor and its concentration was critical, as it directly influences the smoothness and signal-to-noise ratio of the resulting ratio spectra. Divisor concentrations were systematically optimized by evaluating five levels (4, 6, 7, 8, and 10 µg/mL) for each divisor MEL for SAX, and SAX for MET/MEL. These levels were assessed based on spectral noise (baseline SD), peak stability (% amplitude variation), recovery accuracy (*n* = 3 replicates), and %RSD. Lower concentrations (4–6 µg/mL) increased noise and spectral distortion, resulting in recoveries below 98% and %RSD above 2.5. Higher concentrations (10 µg/mL) compressed signals and reduced sensitivity (S/N < 120). The selected concentrations 8 µg/mL MEL for SAX and 7 µg/mL SAX for MET/MEL minimized noise (< 0.004 AU), stabilized peaks (± 0.8% variation), achieved recoveries of 99.2–100.8% with %RSD < 1.4, and provided optimal signal-to-noise ratios (> 160), consistent with standard ratio spectrophotometry practices. Among the tested options, using 8 µg/mL MEL as the divisor for SAX produced the most linear amplitude concentration relationship and the least spectral distortion (Fig. 3), suggesting favorable spectral compatibility between their molar absorptivities. Conversely, for the quantification of MET and MEL, the 7 µg/mL SAX spectrum (Fig. 4) produced the most uniform ratio spectra, allowing clearer discrimination of each component. Wavelength pairs were not selected arbitrarily but were determined by examining ratio spectra for regions where each analyte exhibited a marked amplitude change, while the coexisting components showed nearly constant signals. This ensured that the measured amplitude differences corresponded solely to analyte concentration. The pairs 243.2 and 253.8 nm (SAX), 220.7 and 234.6 nm (MET), and 206.9 and 220.0 nm (MEL) offered the highest sensitivity and selectivity, corresponding to regions of maximum slope change in their respective ratio spectra.

#### First derivative of the ratio spectra spectrophotometric method

**Fig. 5:**
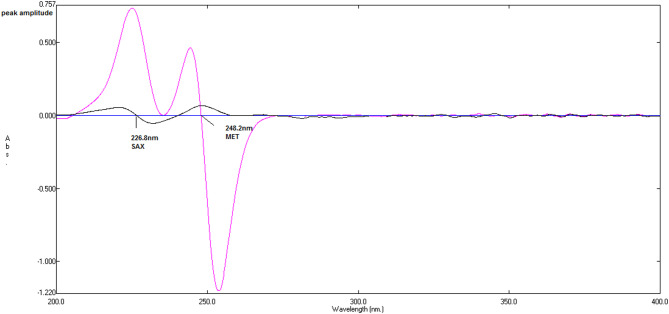
First‑derivative ratio spectra of saxagliptin (SAX, 7 µg/mL) and metformin HCl (MET, 10 µg/mL) obtained by differentiating their ratio spectra after division by the spectrum of melamine (MEL, 5 µg/mL) using water as solvent. The figure illustrates the enhanced resolution and minimized overlap achieved in the first‑derivative ratio domain.

The optimization of Δλ, scaling factor, divisor concentration, and wavelength selection was guided by the spectral characteristics of SAX and MET, rather than predetermined instrumental settings. Various Δλ values (2, 4, and 8 nm) and scaling factors (10, 100, and 1000) were tested. Moderate Δλ values provided an optimal balance between noise suppression and peak resolution, while intermediate scaling factors maintained linearity and baseline stability without amplifying noise. The use of a MEL standard at an appropriate concentration as the divisor produced ratio spectra with a stable baseline and minimal curvature, confirming signal proportionality between the analyte and the divisor and thereby improving method selectivity. Wavelengths were selected in regions where each analyte exhibited a distinct, concentration-dependent amplitude change, while the contribution from the coexisting component was negligible. This ensured well-resolved, diagnostically useful signals in the ratio spectra. For the determination of SAX and MET, a standard spectrum of 5 µg/mL MEL was used as the divisor with Δλ = 4 nm and a scaling factor of 10. The peak amplitudes of the resulting first-derivative ratio spectra were measured at 226.8 nm for SAX and 248.2 nm for MET, as shown in Fig. 5.

### Method validation

Validation results confirm the method’s high reliability for routine quality control analysis. Linear calibration models were established across practical ranges (SAX: 5–90 µg/mL; MET: 1–40 µg/mL; MEL: 0.5–10 µg/mL), with correlation coefficients (r) ≥ 0.9998 and minimal intercept bias, indicating robust linearity. Accuracy was evaluated for both proposed methods by analyzing synthetic mixtures of the drugs at three concentration levels (80%, 100%, and 120% of the nominal target concentration). The mean percent recovery (± SD) was calculated for each analyte. Ratio difference method: the mean recoveries were 100.84 ± 1.19% for SAX, 99.49 ± 1.41% for MET, and 99.43 ± 0.98% for MEL. First derivative of ratio spectra method: The mean recoveries were 101.56 ± 3.79% for SAX and 101.30 ± 3.07% for MET. The recovery results, all close to 100% across the three concentration levels, confirm the high accuracy of the developed methods. Precision was equally satisfactory, with repeatability (intra-day) showing RSD values of 0.36–1.27% and intermediate precision (inter-day) yielding RSD values of 0.36–1.04%. These results demonstrate consistent analyte recovery and minimal variability, ensuring reproducible performance under typical laboratory conditions. Selectivity was verified by the spectral resolution of MET and SAX signals, with no observed interference from tablet excipients. Robustness testing confirmed that minor variations in analytical conditions had a negligible impact on the results. Sensitivity assessment: ratio-difference method LOD: SAX (1.50 µg/mL), MET (0.31 µg/mL), MEL (0.15 µg/mL), first-derivative ratio method LOD: SAX (1.41 µg/mL), MET (0.31 µg/mL), LOQ values: SAX (4.54–4.79 µg/mL), MET (0.94–0.96 µg/mL), MEL (0.45 µg/mL).These sensitivity values support the method’s practical utility for pharmaceutical formulations. Collectively, the validation data establish the procedures as analytically sound, stable, and compliant with ICH Q2(R1)^32^ guidelines for validated spectrophotometric methods.

#### Linearity

Linear relationships for SAX, MET, and MEL were established under the optimized experimental conditions over the concentration ranges of 5–90 µg/mL, 1–40 µg/mL, and 0.5–10 µg/mL, respectively. Each concentration level was analyzed in duplicate (Table 1). High correlation coefficients and low intercept values confirmed excellent linearity.

**Table 1:** Regression and validation parameters for the determination of Saxagliptin, Metformin and Melamine by spectrophotometric methods.

	Ratio difference spectrophotometric method			First derivative of ratio spectra spectrophotometric method	
Parameters	SAX	MET	MEL	SAX	MET
Calibration range	5–90 µg/mL	1–40 µg/mL	0.5–10 µg/mL	5–90 µg/mL	1–40 µg/mL
Slope	0.0058	0.8849	0.8575	0.0004	0.0051
Intercept	0.3042	0.6708	0.4162	0.0009	0.0032
Correlation coefficient (r)*	0.9998	0.9998	0.9999	0.9999	0.9999
Accuracy (Mean ± SD)**	100.84	99.49	99.43	101.56	101.30
Repeatability (%RSD)***	0.51	0.644	0.540	0.86	1.27
Intermediate precision (%RSD)***	0.36	1.04	0.726	0.65	0.58
LOD****	1.50	0.31	0.15	1.41	0.31
LOQ****	4.79	0.96	0.45	4.54	0.94

*Calibration : *n* = 6 points (standard practice).

**Accuracy expressed as mean percent recovery : *n* = 9 (three levels with three replicates each).

***(RSD, %) the intra- and interday relative standard deviation of three different concentrations of Saxagliptin, metformin and melamine.

****Limit of detection and quantitation are determined via calculations (LOD = 3.3 × SD of the response/slope, LOQ = 10 × SD of the response/slope).

#### Accuracy

The accuracy of the proposed methods was evaluated by determining the percentage recovery of pure drug samples. The concentrations, calculated using the corresponding regression equations, are presented in Table 1. Accuracy was further verified using a standard addition approach applied to a commercial MET/SAX formulation (Kombiglyze XR® tablets). The resulting recovery percentages are shown in Table 2 and indicate negligible interference from tablet excipients.

**Table 2:** Recovery results of determination of saxagliptin and metformin in Kombiglyze XR® tablets by the proposed spectrophotometric methods.

	SAX				MET			
	Taken (μg/mL)	Found (% ± SD)	Standard addition		Taken (μg/mL)	Found (% ± SD)	Standard addition	
			Pure added** (μg/mL)	Recovery%			Pure added** (μg/mL)	Recovery%
	Ratio difference spectrophotometric method							
Kombiglyze XR® tablets	(0.1µg mL^-1^ Dosage form + 5 µg/mL pure*	101.2 ± 1.66	7	102.95	20	100.71 ± 0.52	5	102.34
			10	101.37			10	102.01
			20	101.55			20	100.81
			Mean ± SD	101.96 ± 0.86			Mean ± SD	101.72 ± 0.80
	First derivative of ratio spectra spectrophotometric method							
	(0.1µg mL^-1^ Dosage form + 5 µg/mL pure*	99.76 ± 1.49	7	100	20	99.54 ± 2.11	5	98.82
			10	102.5			10	98.43
			20	101.25			20	99.21
			Mean ± SD	101.25 ± 1.25			Mean ± SD	98.82 ± 0.39

*The ratio exists in Kombiglyze XR® tablets prepared by spiking method where 5.1 μg/mL of SAX is (0.1 µg/mL Dosage form + 5 µg/mL pure).

**Standard addition (pure‑added) average of 3 determination.

#### Precision

The intermediate precision and repeatability of the developed methods were evaluated. Repeatability (intra-day precision) was assessed by analyzing three concentrations of pure SAX (5, 15, and 20 µg/mL) and MET (1, 3, and 5 µg/mL) in triplicate (*n* = 3) on the same day. Intermediate precision (inter-day precision) was determined by repeating the same analysis on three consecutive days. The results were satisfactory, with all relative standard deviation (%RSD) values within the acceptable limit of < 2% (Table 1).

#### Limits of detection and quantification (LOD and LOQ)

The sensitivity of the proposed methods was evaluated by determining the limit of detection (LOD) and limit of quantification (LOQ). LOD and LOQ were calculated from the lower linear range of each calibration curve using the formulas LOD = 3.3σ/S and LOQ = 10σ/S, where σ is the standard deviation of the response and S is the slope of the calibration curve.The resulting values, summarized in Table 1, confirm the high sensitivity of the developed techniques. For the ratio-difference method, the LOD values were 1.50, 0.31, and 0.15 µg/mL for SAX, MET, and MEL, respectively, with corresponding LOQs of 4.79, 0.96, and 0.45 µg/mL. For the first-derivative ratio method, the LOD values for SAX and MET were 1.41 and 0.31 µg/mL, respectively, with LOQs of 4.54 and 0.94 µg/mL.

#### Robustness

While the ICH Q2(R1) guideline requires a robustness evaluation during method development to confirm reliability against minor deliberate variations, this assessment is often omitted for sensitive ratio and derivative UV methods. To address this gap, targeted robustness testing was conducted. Key parameters—including wavelength (± 1 nm from λmax), divisor concentration (± 10%), Δλ (± 1 nm), and scaling factor—were systematically varied. Each condition was analyzed with at least three replicates. The method demonstrated excellent stability, as shown by relative standard deviation (RSD) values below 2% for all tested variations (Table 3).

**Table 3:** Robustness evaluation of the proposed spectrophotometric methods (results expressed as %RSD)*

Robustness parameter tested	Ratio difference spectrophotometric method. %RSD results for each component			First derivative of ratio spectra spectrophotometric method. %RSD results for each component	
	SAX	MET	MEL	SAX	MET
Wavelength ± 1 nm	1.12	1.31	1.54	1.101	0.94
Divisor ± 10%	0.9	1.01	1.22	0.22	0.71
Δλ ± 1	Not Reported			0.65	1.47
scaling factor	Not Reported			1.28	0.35

*The robustness was assessed by introducing small, deliberate variations to the method parameters. All %RSD values were within the pre-defined acceptance limit of ≤ 2.0%, confirming the method’s robustness.

#### Specificity

The specificity of the proposed spectrophotometric methods was assessed using laboratory-prepared mixtures containing SAX, MET, and MEL in varying proportions across their respective linear ranges (Table 4). At the selected analytical wavelengths, no significant absorbance contribution from the coexisting components was observed, confirming the absence of spectral interference and validating the specificity of the procedures.

**Table 4:** Determination of Saxagliptin, Metformin and Melamine in laboratory prepared mixtures by the spectrophotometric methods.

Mix no	Ratio difference spectrophotometric method						First derivative ratio spectrophotometric method			
	SAX		MET		MEL		SAX		MET	
	Taken µg/mL	% Recovery**	Taken µg/mL	% Recovery**	Taken µg/mL	% Recovery**	Taken µg/mL	% Recovery**	Taken µg/mL	% Recovery**
1	5.1*	98.71	20	102.98	0.5	98.68	5.1*	98.03	20	99.21
2	30	98.96	10	102.80	1	98.04	30	99.16	10	98.43
3	40	99.65	20	98.14	2	100.05	40	99.37	20	98.23
4	30	98.39	20	97.05	0.5	99.42	30	99.16	20	100.19
5	20	99.31	30	99.62	5	98.46	20	101.25	30	98.16
6	20	98.44	25	102.70	4	99.41	20	100	25	98.19
Mean ± SD	98.91 ± 0.49		100.53 ± 2.61		99.01 ± 0.73		99.49 ± 1.06		98.74 ± 0.8	
%RSD	0.50		2.60		0.74		1.07		0.81	

*The ratio exists in Kombiglyze XR® tablets prepared by spiking method where 5.1 µg/mL of SAX is (0.1 µg/mL Dosage form + 5 µg/mL pure).

**Each reported % Recovery value is the mean of three determinations.

### Analysis of laboratory-prepared mixtures

Laboratory-prepared mixtures of SAX, MET, and MEL were constructed at various ratios and concentrations within their linear ranges using the corresponding working standard solutions. The UV spectra of these mixtures were recorded over the 200–400 nm range. The concentration of each drug in the mixtures was determined using its respective regression equation. Table 4 presents the results, including those for mixture 1, in which the SAX level was adjusted via a spiking procedure to achieve the target pharmaceutical formulation ratio of 5 µg/mL SAX to 1000 µg/mL MET.

### Application of pharmaceutical formulation

This study evaluated the performance of the developed spectrophotometric methods for quantifying SAX and MET in Kombiglyze XR® tablets to assess their suitability for commercial formulation analysis. Recovery studies were performed using a standard addition approach, where known quantities of pure SAX and MET standards were spiked into pre-analyzed tablet solutions. Each experiment was conducted in triplicate (*n* = 3) to ensure statistical reliability. The results confirmed the absence of significant interference from tablet excipients. The validity of the proposed methods was further supported by recovery studies conducted using the standard addition approach, as shown in Table 2. The results obtained from the analysis of SAX and MET in the pharmaceutical formulation by the proposed methods were statistically compared with those from a reported RP-HPLC method^19^ using Student’s t-test and the variance ratio F-test. At the 95% confidence level, no significant differences were observed between the two methods, all calculated t-values (0.031–2.22) (0.211–0.12) for SAX and MET respectively were below the critical value of 2.228 (df = 10), and all F-values (1.005–1.39) (1.029–1.11) for SAX and MET respectively were below the critical value of 5.050 (df = 5,5). This confirms the absence of significant differences in means or variances (*p* > 0.05). The statistical equivalence of the proposed spectrophotometric methods to the reference HPLC method is demonstrated in Table 5 and is consistent with ICH Q2(R1) validation requirements^32^.

**Table 5:** Statistical comparison of the results obtained by the proposed method and the reference methods.

Method	Ratio difference spectrophotometric method		First derivative of ratio spectra spectrophotometric method		Reported method*	
Items	SAX	MET	SAX	MET	SAX	MET
Mean	101.2	100.71	99.76	99.54	102.61	101.18
%RSD	1.66	0.52	1.49	2.11	1.69	0.94
Variance	3.22	0.20	2.83	4.80	2.87	0.88
n	6	6	6	6	6	6
Student’s t-test (2.228)** df = (10)***	0.031	0.211	2.22	0.12	–	–
F-value (5.050)** _df=(5.5)_***	1.005	1.029	1.39	1.11	–	–

*The results obtained from a previously disclosed RP-HPLC method^19^ was conducted using the Student’s t-test and the variance ratio F-test.

**The tabulated (critical) t- and F-values.

***Degree of freedom, formulas are (df = n_1_ + n_2_–2) for the t-test and (df = n–1) for each variance in the F-test.

### Greenness assessment

Green analytical metrics confirmed that the developed spectrophotometric methods are environmentally benign and resource-efficient. The use of water as a non-PBT (Persistent, Bio accumulative, Toxic) solvent, coupled with the generation of less than 50 mL (or 50 mg) of waste which refers to the estimated waste generated per analytical run (per sample analysis) rather than the total waste from all experiments, fulfilled all four quadrants of the national environmental methods index (NEMI)^40^, supporting their classification as green methods. The green analytical procedure index (GAPI)^41^ assessment yielded a pictogram with five green zones (indicating best practices), eight yellow zones (indicating moderate environmental impact), and two red zones (indicating areas for improvement). This result reflects a generally favorable greenness profile with only minor aspects requiring optimization. The analytical greenness (AGREE) metric^42,43^, which evaluates compliance with the 12 principles of green analytical chemistry, produced an overall score of 0.81 (on a 0–1 scale, where 1 is ideal). This high score, visualized in a circular pictogram, indicates that the method excels in key environmental aspects such as solvent choice (water), low solvent volumes, minimal waste generation, and low toxicity. Only minor elements prevented a near‑ideal rating. The complete greenness assessment data are summarized in Table 6.

**Table 6:**
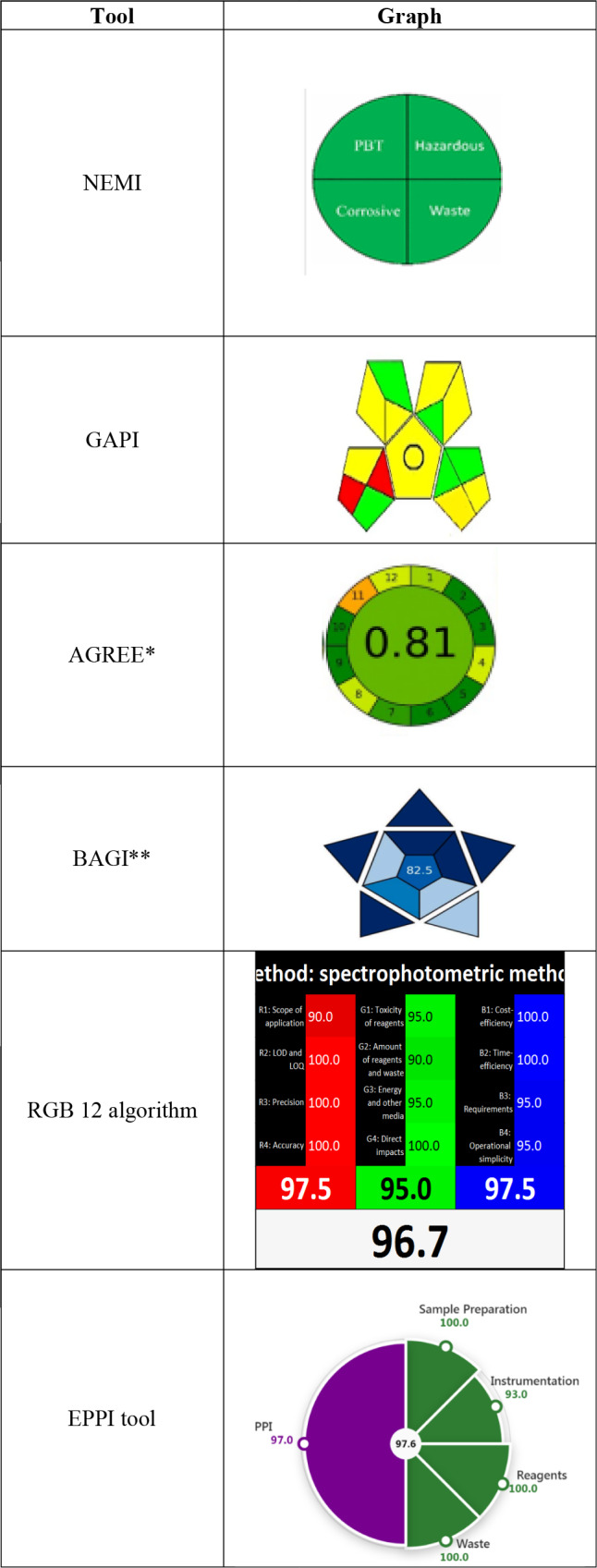
Greenness and blueness assessment of the suggested spectrophotometric methods.

*The AGREE score of 0.81 (scale 0–1) indicates the method has a high level of analytical greenness.

**The BAGI score of 82.5% (scale 0–100%) reflects a strong overall performance of the analytical method.

### Blueness evaluation

The blue applicability grade index (BAGI)^44^ assesses the practical performance of an analytical method across ten distinct criteria, presenting the results as an asteroid-shaped pictogram with a central overall score. A final score above 60% indicates a practically useful method. The proposed spectrophotometric methods achieved an overall score of 82.5%, directly reflects the practical strengths of the proposed method, which is well-suited for routine use. These strengths include the use of a common and economical UV–Vis spectrophotometer, minimal and simple sample preparation, rapid analysis enabling high throughput, and the eco-friendly and cost-effective choice of water as the solvent. Furthermore, the method consumes less energy than chromatographic techniques and is founded on an efficient, mathematically-driven approach for analyzing multiple components without physical separation. Collectively, these attributes confirm the method’s strong applicability and readiness for implementation in quality-control laboratories. (Table 6).

### White analytical chemistry

The RGB 12 algorithm^45^, introduced in 2021, refines the original RGB metric to provide a comprehensive assessment of analytical methods. It evaluates 12 criteria that determine whether an assay aligns with the goals of green and “white” analytical chemistry. These criteria are organized into three categories: red (analytical quality), the high Red score confirms the method’s analytical robustness, excelling in accuracy (~ 100% recovery, low RSD), selectivity (resolving SAX, MET, and MEL despite overlap), sensitivity (low LOD/LOQ), and reliability (wide linear range and proven robustness), green (environmental and safety impact), the high Green score validates the method’s environmental sustainability. Its key advantages include the use of water as a benign solvent, which eliminates hazardous organic waste; high energy efficiency compared to chromatographic techniques; generation of minimal non-toxic aqueous waste; and enhanced laboratory safety due to the use of non-flammable, non-toxic reagents. And blue (practical and economic feasibility), the high Blue score reflects the method’s strong practical and economic feasibility for routine use. It is underpinned by the widespread availability and low cost of UV–Vis instrumentation, straightforward operational procedures that require minimal training, rapid analysis enabling high sample throughput, and a very low cost per analysis due to inexpensive reagents and minimal operating expenses. The integrated scores are calculated within a dedicated spreadsheet, yielding an overall RGB12 value. The proposed spectrophotometric method achieved a combined overall score of 96.7%, confirming its simultaneous excellence in analytical performance, greenness, and practical applicability, and its strong suitability for sustainable routine analysis (Table 6).

### EPPI tool

The environmental index (EI) evaluates an analytical method’s environmental sustainability based on the principles of green analytical chemistry (GAC) and green separation processes (GSP). It assesses four key elements: sample preparation (S), instrumentation (I), reagent type and quantity (R), and waste production (W). The proposed method achieved an EI score of 98.3%, placing it in the ideal green range (85–100%). This high score reflects its minimal sample preparation, efficient spectrophotometric instrumentation, use of low-toxicity reagents, and reduced waste generation, clearly outperforming less sustainable alternatives (EI < 55%).

The practical and performance index (PPI) assesses method applicability across critical parameters, including method type, quality by design (QbD) integration, validation scope, sensitivity, reagent availability and cost, analysis cost, instrument availability, maintenance requirements, throughput, and sample reusability^46^. These parameters collectively gauge a method’s viability for routine, high-volume use, emphasizing efficiency, affordability, and operational flexibility. The proposed method achieved an excellent PPI score of 97.0% (where 75–100% is considered excellent) Table 6.

### Comparing the developed techniques with the spectrophotometric method that was previously published

Table 7 highlights the significant advantages of the proposed spectrophotometric methods over a previously reported method^10^ for the simultaneous determination of saxagliptin (SAX) and metformin (MET). The new methods demonstrate superior sensitivity and selectivity. A key advancement is their capability to accurately quantify both analytes in the presence of melamine (MEL), a hazardous impurity of metformin. Furthermore, the environmental friendliness and practical utility of the proposed methods were rigorously evaluated using established green analytical chemistry metrics an assessment that was absent in the prior literature.

**Table 7:** A comparison between the developed methods and previously published spectrophotometric method.

Parameters	Developed methods [Ratio difference& First derivative of ratio spectra spectrophotometric method]		Previously published method* [Zero order spectrophotometric method]	
Component	SAX	MET	SAX	MET
Linearity	5–90 µg/mL	1–40 µg/mL	50–90 µg/mL	2–10 µg/mL
LOD	1.50 µg/mL 1.41 µg/mL	0.31 µg/mL 0.31 µg/mL	7.32 µg/mL	1.2 µg/mL
LOQ	4.79 µg/mL 4.54 µg/mL	0.96 µg/mL 0.94 µg/mL	21.62 µg/mL	3.6 µg/mL
Sensitivity comparison***	LOD ratio:			
	SAX = 5.0x	MET = 3.9x		
	LOQ ratio:			
	SAX = 4.6x	MET = 3.8x		
Analytes	SAX, MET, MEL		SAX, MET only	
Impurity Handling	Accurately quantifies MEL (hazardous impurity)		No MEL inclusion	
Greenness assessment	--------- NEMI GAPI AGREE		Not reported	
Blueness assessment	BAGI		Not reported	
White analytical chemistry	RGB 12 algorithm		Not reported	
EPPI	Evaluated		Not reported	

*The results obtained from a previously disclosed spectrophotometric method^10^.

**LOD/LOQ values for the developed methods are presented as the mean of the two reported techniques (Ratio Difference and First Derivative of Ratio Spectra), LOD& LOQ ratio (Published/Developed).

***Sensitivity is quantified by the fold-improvement in LOD and LOQ. A ratio > 1 indicates superior sensitivity of the developed methods.

## Comparison between developed methods and previously published chromatographic method

A comparative greenness assessment (Table 8) shows that the developed spectrophotometric methods perform equivalently to or better than reported UPLC and HPTLC methods across key metrics. All three methods fulfill all NEMI criteria (four green quadrants). Their GAPI profiles are similar, with the developed method having 5 green, 8 yellow, and 2 red zones compared to 5 green, 7 yellow, and 2 red for the others, indicating comparable environmental impacts from solvent use and waste generation. In the AGREE assessment, the spectrophotometric methods score well (0.81), ranking between the UPLC (0.86) and HPTLC (0.77) methods. Furthermore, the proposed methods were uniquely evaluated using the BAGI, RGB12, and EPPI tools, yielding scores that underscore their practical utility and environmental excellence: a BAGI score of 82.5% (indicating good practicality), an RGB12 score of 96.7%, and an EPPI score with an environmental index (EI) of 98.3% and a practical performance index (PPI) of 97.0%. These comprehensive evaluations, not reported for the earlier methods, confirm the overall eco‑superiority and strong suitability for routine laboratory use in the ternary analysis of SAX, MET, and MEL^47^.

**Table 8:** A comparison between developed methods and previously published chromatographic method.

Metric	Developed methods	Previously published UPLC method*	Previously published HPTLC method*
NEMI	Meets the acceptance criteria for all four quadrants	Meets the acceptance criteria for all four quadrants	Meets the acceptance criteria for all four quadrants
GAPI	Yielded 5 green, 8 yellow, and 2 red zones	Yielded 5 green, 7 yellow, and 2 red zones	Yielded 5 green, 7 yellow, and 2 red zones
AGREE	Assigned an overall score of 0.81	assigned an overall score of 0.86	Assigned an overall score of 0.77
BAGI	Overall score of 82.5	Not evaluated	Not evaluated
RGB 12 algorithm	Overall score of 96.7	Not evaluated	Not evaluated
EPPI	Overall score of (EI) 98.3% With a PPI score of 97.0%	Not evaluated	Not evaluated

*The results obtained from a previously chromatographic method^31^.

## Conclusion

This work presents simple, rapid, and cost-effective spectrophotometric methods for the simultaneous quantification of saxagliptin (SAX), metformin (MET), and the hazardous impurity Melamine (MEL). The ratio-difference and first-derivative of ratio spectra techniques successfully quantified SAX and MET in their pure forms and in a commercial tablet formulation (Kombiglyze XR®), even in the presence of MEL and despite the challenging 200:1 (MET:SAX) concentration ratio. The environmental friendliness of the methods was rigorously validated using established green metric tools (NEMI, GAPI, and AGREE). Their practical applicability was further confirmed through assessments with the blue applicability grade index (BAGI), the RGB 12 algorithm, and the environmental and practical performance index (EPPI). Due to their accuracy, eco-friendliness, and cost-effectiveness including minimal solvent use, low energy consumption, and reduced waste generation these methods are suitable for routine quality control (QC) applications for routine pharmaceutical analysis.

## Supplementary Information

Below is the link to the electronic supplementary material.

Supplementary Material 1

## Data Availability

All data generated or analysed during this study are included in this published article [and its supplementary information files].
