# Supplementary material for: Eco-friendly spectrophotometric approach for the determination of anti-diabetic drugs in fixed-dose formulation together with metformin’s toxic impurity: comprehensive method assessment
Source: Sci Rep. 2026 Mar 21;16:9687. doi: 10.1038/s41598-026-38952-3 (PMC13009257; doi:10.1038/s41598-026-38952-3)
Supplement: Supplementary file 1 — Supplementary Material 1 [file 41598_2026_38952_MOESM1_ESM.docx]

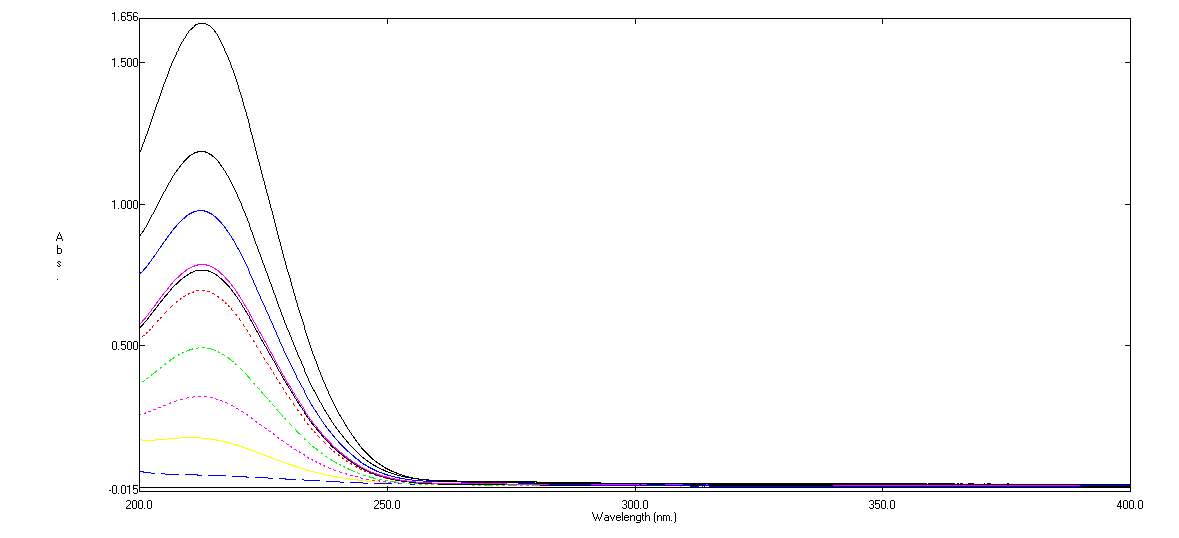


S .1. Zero-order UV spectra of saxagliptin (5–90 µg/mL) in Water (200–400 nm), demonstrating linear absorbance at λ_max 212 nm for quantitative determination.


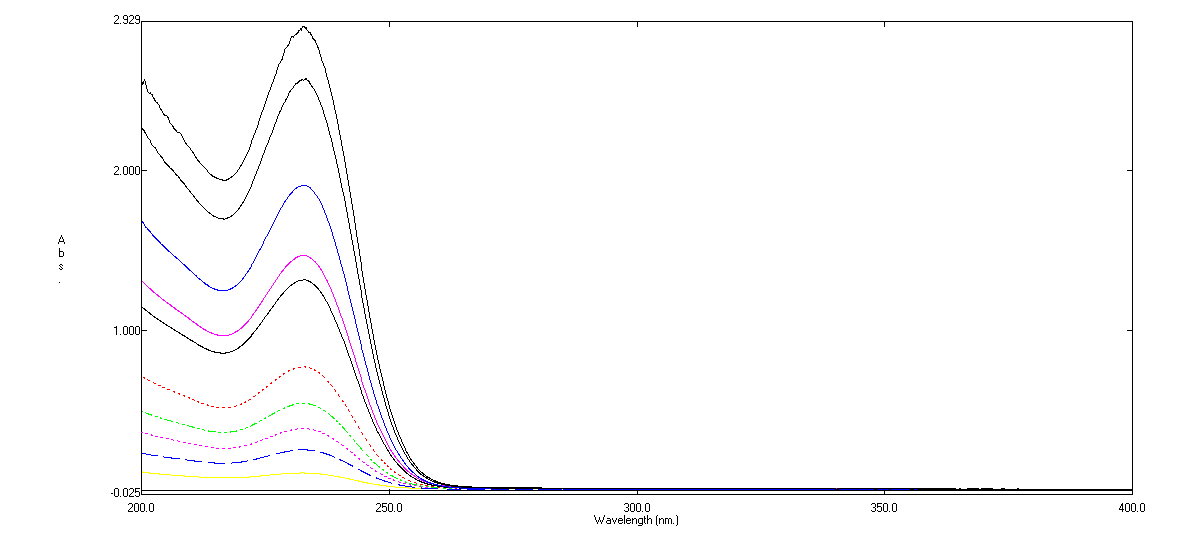


S .2. Zero-order UV spectra of metformin (1–40 µg/mL) in Water (200–400 nm), demonstrating linear absorbance at λ_max 233 nm for quantitative determination.


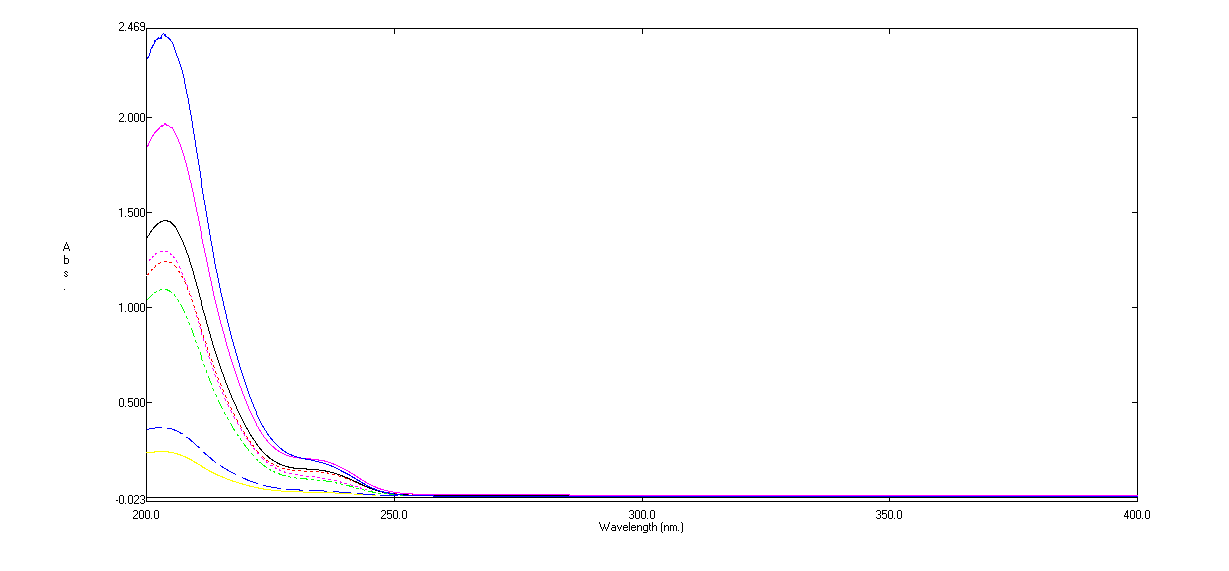


S .3. Zero-order UV spectra of melamine (0.5-10 µg/mL) in Water (200–400 nm), demonstrating linear absorbance at λ_max 203 nm for quantitative determination.
